# Monocular Visual Pose Estimation Method Based on Spherical Cooperative Target

**DOI:** 10.3390/s26103139

**Published:** 2026-05-15

**Authors:** Yanyu Ding, Chaoran Zhang, Yongbin Zhang, Fujin Yang, Zhiyuan Tang, Shipeng Li, Xinran Liu, Xiaojun Zhao

**Affiliations:** 1 School of Mechanical Engineering, Tianjin University of Science and Technology, Tianjin 300457, China; dingyanyu@tust.edu.cn (Y.D.); 17866919877@163.com (F.Y.); lxr512807@163.com (X.L.); liebesteaks@gmail.com (X.Z.); 2 Tianjin Key Laboratory of Integrated Design and Online Monitoring for Light Industry & Food Machinery and Equipment, Tianjin University of Science and Technology, Tianjin 300457, China; 3 Tianjin Navigation Instrument Research Institute, Tianjin 300131, China; 4 School of Mechanical Engineering, Tianjin University, Tianjin 300354, China

**Keywords:** spherical target, monocular visual measurement, pose estimation, PnP, stereo triangulation, nonlinear optimization

## Abstract

In close-range monocular visual measurement and cooperative target pose estimation, conventional planar targets are constrained by viewpoint changes and are prone to perspective distortion. Although spherical targets provide omnidirectional observability, their PnP-based pose estimation may still suffer from large errors under limited fields of view and sparse feature observations. To address this issue, this paper proposes an integrated visual measurement framework covering both high-precision spherical target construction and robust pose estimation. First, a composite marker layout based on adaptively scaled latitude–longitude topology is designed. To suppress cumulative distortion caused by long-sequence multi-view rigid registration, a center-to-pole point-cloud stitching strategy is developed, and multiple observations are fused using geometric-consistency weighting to accurately reconstruct the feature-point coordinate system of the target. Second, a joint optimization method is proposed by combining feature-point reprojection error with a contour center consistency constraint. Specifically, the theoretical contour center is predicted from the analytical projection model of the sphere and constrained to agree with the observed contour center fitted from the image. In addition, an SQPnP-based sequential reinitialization mechanism is introduced to improve robustness under sparse-point observations. Simulation results demonstrate that the proposed method achieves higher accuracy and robustness under continuous pose changes, sparse feature points, and different noise levels, compared with EPnP, EPnP+LM, LM, and SQPnP, while real-image experiments further demonstrate its practical feasibility.

## 1. Introduction

In robotic visual measurement, cooperative target localization, and monocular pose estimation, recovering the pose of a target in a stable and accurate manner remains a fundamental challenge in computer vision [1,2,3]. Most existing methods establish 2D–3D correspondences based on artificial cooperative targets or natural image features and formulate pose estimation as a Perspective-n-Point (PnP) problem [4,5,6]. Although these methods are mature and widely used, their performance can deteriorate significantly under challenging conditions, particularly when the number of feature points is insufficient, their spatial distribution becomes degenerate, or image noise is severe. This limitation is especially evident in continuous pose-change scenarios, where methods relying solely on sparse discrete feature points are highly sensitive to feature extraction quality, correspondence accuracy, and initialization [7,8,9]. Therefore, designing cooperative targets that can provide more stable and informative geometric constraints is essential for improving pose estimation performance under challenging conditions.

Compared to traditional planar targets, spherical targets offer distinct advantages owing to their isotropic geometry and stable closed contour [10,11,12,13]. Early work by Sun et al. employed sphere targets for global calibration of multiple cameras, demonstrating their ability to provide consistent and accurate center estimation across different viewpoints [14]. In recent years, Jiang et al. developed a ball-shaped target for tracking-based scanning systems and proposed a corresponding pose estimation and tracking strategy, achieving improved robustness in dynamic measurement scenarios [15]. Tóth et al. presented a minimal solution for image-based sphere estimation that exploits the isotropy of spherical targets, requiring only three contour points to accurately fit the projected ellipse and estimate sphere parameters, thereby enhancing efficiency and numerical stability [16].

These studies suggest that the intrinsic geometric properties of spherical targets offer stable and viewpoint-invariant geometric constraints, which can be effectively leveraged to enhance pose estimation robustness. Furthermore, by rationally distributing discrete markers across the spherical surface, the target can simultaneously utilize the sphere’s stable geometric constraints and local feature point information, ensuring accurate PnP pose estimation even in scenarios with sparse feature points.

Motivated by these observations, this study designs a spherical feature-point layout based on a latitude–longitude layered arrangement and develops a composite marker unit consisting of concentric rings and ArUco codes to ensure both uniform point distribution and sub-pixel localization accuracy. On this basis, a stereo-vision-based method is further proposed to construct the 3D feature-point model of the spherical target. Through multi-view local point-set reconstruction, rigid registration between adjacent point sets, and iterative optimization using multi-frame observations, a global 3D feature-point model centered at the sphere is obtained, providing a unified spatial reference for subsequent pose estimation.

For pose estimation, numerous PnP methods have been developed for different application scenarios. For example, the EPnP (Efficient PnP) method proposed by Lepetit et al. introduces virtual control points to transform the original nonlinear problem into a linear system, enabling fast initial pose estimation [17]. The SQPnP (Stable and Quick PnP) method proposed by Terzakis and Lourakis formulates the problem within a globally optimal framework, improving robustness under noisy and degenerate configurations while maintaining high computational efficiency [18]. More recently, Qiao et al. proposed Hidden PnP, which parameterizes the rotation matrix using hidden variables and refines the solution via Gauss–Newton iterations, achieving improved accuracy and robustness under noise for general 3D, planar, and near-singular configurations [19]. Henry and Christian proposed the optimal DLT (oDLT) method, which provides a near-optimal non-iterative solution using weighted normalized direct linear transformation. Its performance is comparable to Gauss–Newton optimization while requiring significantly lower computational cost, and it demonstrates improved stability and efficiency under noisy and narrow-field-of-view conditions [20]. Despite the effectiveness of these methods under standard conditions, pose estimation in complex industrial environments may still suffer from large errors or convergence to local minima due to noise, limited visible feature points, and other adverse factors. Therefore, further optimization of pose estimation strategies remains necessary for practical applications.

**Figure 1:**
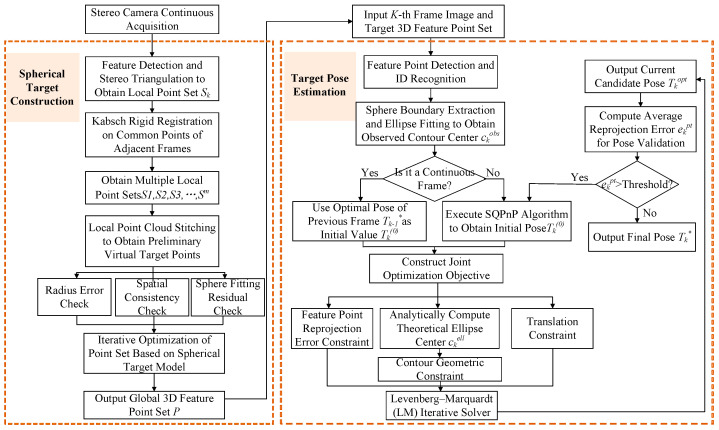
Overall flowchart of the proposed spherical target construction and pose estimation algorithm, where * denotes a symbolic marker.

To address the above issues, this paper proposes a robust joint optimization method for PnP based on spherical analytical geometric priors. First, local marker point sets on the spherical surface are reconstructed via stereo triangulation, and a unified coordinate system is established through registration of multiple local point sets. During pose estimation, the reprojection error of feature points is used as the primary constraint, while a consistency constraint between the observed contour center and the theoretical contour center is introduced to form a joint optimization model. To improve the stability of continuous pose estimation, the first frame is initialized using SQPnP, and the optimal pose of the previous frame is used as the initial estimate for iterative refinement of the current frame. In addition, Huber-based robust optimization and a reprojection-error-threshold-based reinitialization mechanism are incorporated to achieve stable and continuous pose estimation. The overall workflow is illustrated in Figure 1.

## 2. Spherical Coded Target and Structural Design

### 2.1. Spherical Target Design

**Figure 2:**
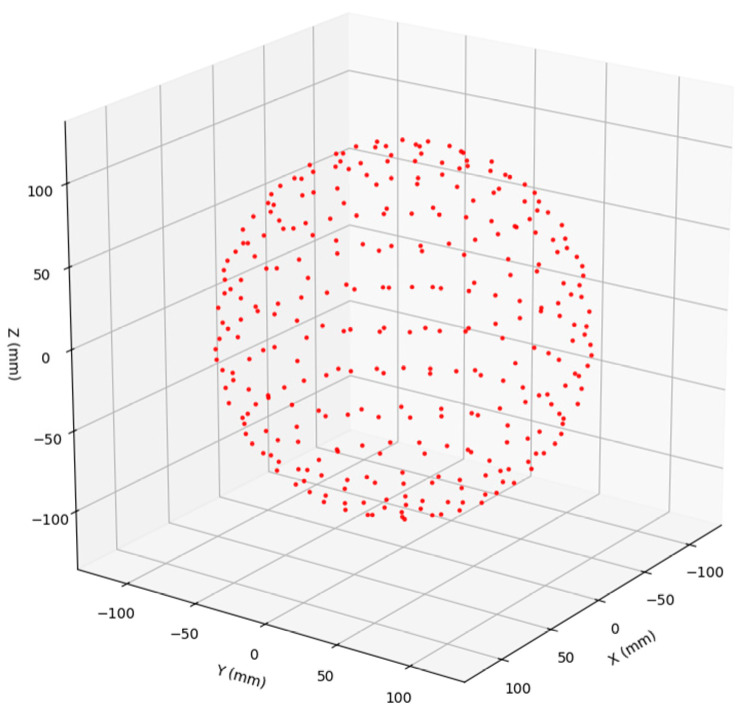
Designed feature point layout based on adaptive latitude–longitude topology on the spherical surface, where discrete red dots represent each feature point of the target.

Accurate PnP-based pose estimation requires at least four visible marker points in each frame. To ensure reliable and well-conditioned global pose estimation of the spherical target, the markers distributed on the spherical surface should be as uniform as possible. In principle, the Fibonacci lattice can achieve approximately uniform sampling on a spherical surface; however, it lacks an explicit structural topology, which is unfavorable for target reconstruction and practical fabrication [21]. Therefore, this study adopts a latitude–longitude layered arrangement with a regular topological structure, which preserves distribution uniformity while providing positional constraints among neighboring markers. The overall point layout is illustrated in Figure 2.

Let the physical radius of the spherical target be R, and let the desired average arc-length spacing between adjacent markers on the spherical surface be d. According to this density requirement, the spherical surface is divided into L latitude layers, and the baseline number of marker points on the equator, denoted by $N_{eq}$, is defined as

(1) $$\begin{array}{cccc} & L=round\left(\frac{\pi R}{d}\right),N_{eq}=round\left(\frac{2\pi R}{d}\right) & \; & \end{array}$$

To avoid excessively high point density near the polar regions, the number of markers deployed in the i-th latitudinal layer (corresponding to the elevation angle $\theta_{i}$), denoted as $N_{i}$, is adaptively scaled according to the circumference of the corresponding latitude circle:

(2) $$\begin{array}{cccc} & N_{i}=round(N_{eq}\cos\theta_{i}) & \; & \end{array}$$

**Figure 3:**
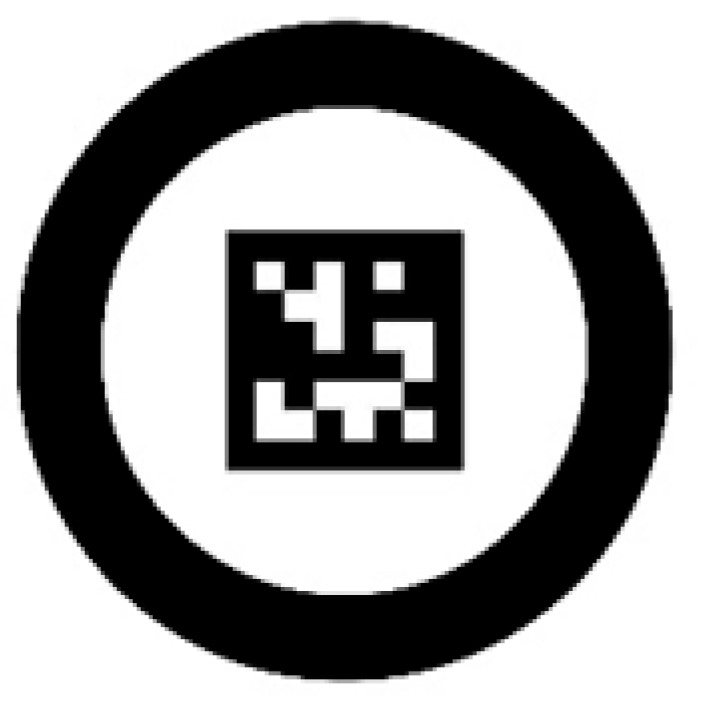
Structure of the proposed composite marker unit.

Despite the established feature distribution, a single type of coded marker is often insufficient to ensure high-precision localization due to spherical curvature and imaging distortion. To address this limitation, a composite marker unit is proposed that integrates coded markers with geometric features. Specifically, the outer layer adopts a concentric ring structure to provide stable sub-pixel center localization, while the inner layer employs an ArUco code to enable unique ID identification. The marker unit is illustrated in Figure 3. By incorporating concentricity error compensation, the positioning accuracy and robustness of feature points under varying viewing angles and distances are significantly improved [22].

In addition, to ensure that the minimum number of feature points required by PnP is satisfied under arbitrary viewpoints, the relationship between the total number of markers and the field-of-view (FOV) range is further established. Let the effective monocular field of view projected onto the target plane have a side length of L, and let the marker ring diameter be d. The effectively observable area within a single view can be approximated as $\left(L-d{)}^{2}\right.$.To guarantee that at least $M_{min}$ feature points are observable, where $M_{min}=4$, the total number of markers on the spherical target should satisfy.

(3) $$N\geq M_{\min}\cdot \frac{4\pi R^{2}}{(L-d{)}^{2}}$$

Taking R=73.8 mm, ring diameter d=4 mm, and $M_{min}=4$ as an example, the minimum required number of markers $N_{min}$ under different field-of-view side lengths L can be calculated. The corresponding results are summarized in Table 1.

### 2.2. Construction of the Global Coordinate System of the Spherical Target

To establish a global coordinate system with the sphere center as the origin and to obtain the 3D coordinates of each marker point $P_{i}=[X_{i},Y_{i},Z_{i}{]}^{T}$, a spherical target construction method based on stereo vision is proposed.

**Figure 4:**
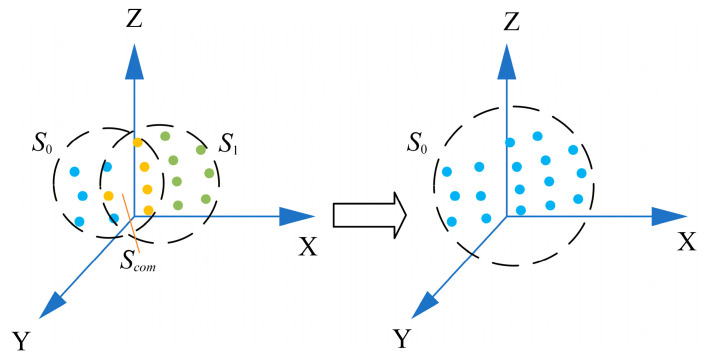
Rigid transformation of a local feature point set between two consecutive frames, where blue dots denote the points of the initial reference set $S_{0}$, yellow dots represent the common points $S_{com}$  between the two point sets, and green dots stand for the points of the second frame point set $S_{1}$ .

First, according to the triangulation principle [23], the 3D coordinates of local marker points are reconstructed from a single viewpoint to form an initial reference point set $S_{0}$. The spherical target is then rotated continuously with small angular increments, and multiple local point sets $S_{k}$ $\left(k=1,2,\ldots \right)$ are sequentially acquired. For each pair of adjacent point sets $S_{k-1}$ and $S_{k}$, their common marker subset $S_{com}$ is extracted, where the number of common points satisfies $N_{com}\geq 3$. As shown in Figure 4, to align point sets from different frames, the optimal rigid transformation between adjacent frames is estimated using the Kabsch algorithm [24]. Specifically, after zero-centering the common point sets, the covariance matrix H is constructed from corresponding points:

(4) $$\begin{array}{cccc} & H=\sum_{j=1}^{N_{com}}\left(P_{j}^{k}-{\bar{P}}^{k}\right){\left(P_{j}^{k-1}-{\bar{P}}^{k-1}\right)}^{T} & \; & \end{array}$$

By performing singular value decomposition of H as $H=U\Sigma V^{T}$, the optimal rotation matrix is obtained as $R_{k}=VU^{T}$, and the translation vector $t_{k}$ is computed from the centroid relationship, thereby completing the rigid alignment between adjacent point sets. The transformation parameters $(R_{k},t_{k})\;$ are obtained by minimizing the sum of squared distances between corresponding points in adjacent frames:

(5) $$\begin{array}{cccc} & (R_{k},t_{k})=arg\;\underset{R,t}{min}\sum_{j=1}^{N_{com}}{∥P_{j}^{k-1}-(RP_{j}^{k}+t)∥}^{2} & \; & \end{array}$$

**Figure 5:**
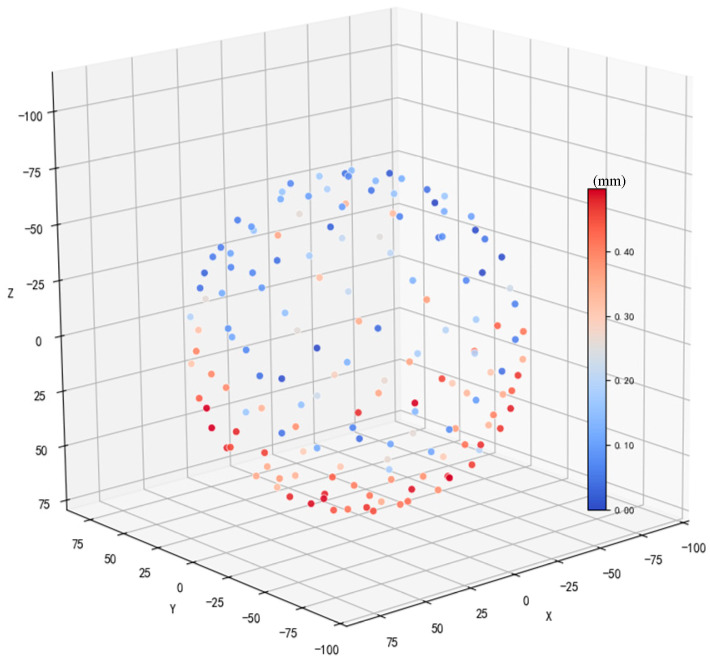
Cumulative error accumulation during spherical target coordinate system construction.

Ideally, repeating the above alignment process would be sufficient to reconstruct the complete point cloud of the spherical target. However, in practical data acquisition, performing registration along a single direction over a long sequence may lead to gradual accumulation of observation noise, resulting in significant errors. Geometrically, such accumulated errors cause the reconstructed point cloud to deviate from the ideal spherical surface. As shown in Figure 5, the point colors from blue to red represent the Euclidean distance between each point and the fitted sphere. When reconstruction starts from one pole, points near the opposite pole tend to exhibit larger errors as the number of accumulated frames increases.

**Figure 6:**
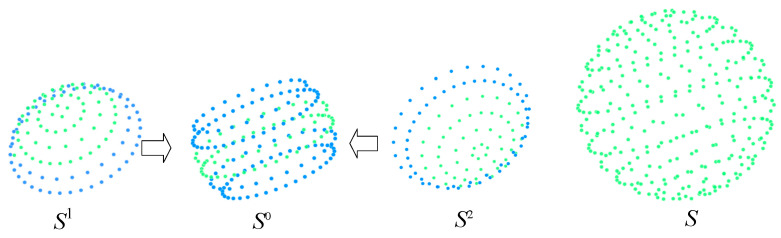
Construction of the initial target point set, where blue points denote common points shared between different reference point sets, and green points denote the points to be registered from different reference point sets.

To mitigate error accumulation, a center-to-pole expansion strategy is adopted for spherical target construction. First, multiple relatively independent reference point sets, denoted as $S^{1},S^{2},S^{3},\ldots ,S^{m}$, are established in different regions of the spherical surface. As shown in Figure 6, starting from the central region, several local point clouds are reconstructed within their respective local coordinate systems, and these local point clouds are then unified into a common global coordinate system through point-cloud registration, yielding an initial complete point set S.

Since each marker (with global ID i) may be observed multiple times during the rotation process, multiple 3D coordinate estimates with slight deviations can be obtained, denoted as

$$P_{i,1},P_{i,2},\ldots ,P_{i,c}$$

To determine the optimal coordinate of each marker, a geometry-consistency-based observation evaluation mechanism is introduced to identify and remove outliers.

For each observed $P_{i,c}$, a comprehensive score is defined as

(6) $$\begin{array}{cccc} & S_{i,c}=\alpha_{1}E_{r}+\alpha_{2}E_{s}+\alpha_{3}E_{c} & \; & \end{array}$$

where $E_{r}=|∥P_{i,c}-O∥-R|\;$ represents the sphere-radius constraint error, $E_{s}$ denotes the deviation of the current observation from the mean of observations of the same marker, and $E_{c}$ measures the contribution of the current point to the overall sphere-fitting residual. The coefficients $\alpha_{1}$, $\alpha_{2}$, and $\alpha_{3}$ are non-negative weights satisfying $\alpha_{1}+\alpha_{2}+\alpha_{3}=1$.

All observations are ranked according to the score function, and those exceeding a predefined threshold are removed, yielding a valid observation set $V_{i}$. Based on this set, the optimal coordinate of the i-th marker is obtained by minimizing the weighted spatial error:

(7) $$\begin{array}{cccc} & P_{i}^{*}=arg\;\underset{P_{i}}{min}\sum_{c\in V_{i}}w_{c}{∥P_{i}-P_{i,c}∥}^{2} & \; & \end{array}$$

The weighting coefficient $w_{c}$ determined jointly by the reprojection error and the geometry-consistency score:

(8) $$\begin{array}{cccc} & w_{c}=exp(-\lambda_{w}e_{c})\cdot exp(-\mu_{w}S_{i,c}) & \; & \end{array}$$

where $e_{c}$ is the reprojection error in the c-th frame, and $\lambda_{w}$ and $\mu_{w}$ are scaling factors controlling the influence of reprojection error and geometric consistency, respectively.

By solving the above optimization, the optimal coordinate corresponds to the weighted centroid of all valid observations. By traversing all marker IDs and applying this optimization procedure, the complete 3D feature-point set P of the spherical target is finally obtained. The reconstruction error statistics of the target feature points are summarized in Table 2.

## 3. Pose Estimation Method Based on Spherical Target

**Figure 7:**
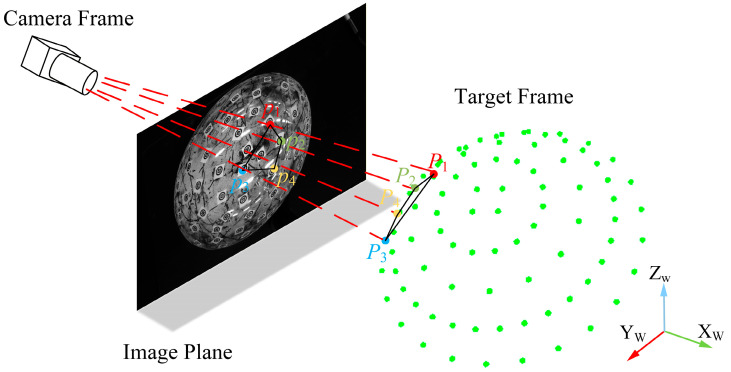
Schematic diagram of pose estimation using the PnP algorithm, where $P_{1}$, $P_{2}$, $P_{3}$  and $P_{4}$  are the corresponding feature points between the 2D image and the 3D target coordinate system in the PnP algorithm.

To achieve stable pose estimation $T_{k}=\{R_{k},{\;t}_{k}\}$ across consecutive frames based on the reconstructed three-dimensional feature points, a robust PnP-based joint optimization method incorporating spherical geometric priors is proposed. The method takes the reprojection error of feature points as the primary objective, while introducing geometric consistency constraints derived from spherical contour projection, as well as a translation reference term to enhance the stability and convergence of sequential pose estimation. Let $S_{k}$ denote the set of successfully matched feature points in the k-th frame. As shown in Figure 7, for a three-dimensional feature point $P_{i}\;$in the target coordinate system with corresponding image observation $p_{i,k}$, the predicted projection under pose $T_{k}$ is given by

(9) $$\begin{array}{cccc} & {\hat{p}}_{i,k}=\pi \;\left(K(R_{k}P_{i}+t_{k})\right) & \; & \end{array}$$

where K denotes the camera intrinsic matrix and π(⋅) represents the perspective projection function from homogeneous coordinates to pixel coordinates.

For numerical optimization, the rotation matrix $R_{k}\;$is parameterized by a Rodrigues vector $r_{k}\in R^{3}$, and the optimization variable is defined as $x_{k}=[r_{k}^{T},t_{k}^{T}{]}^{T}$. In addition to the reprojection constraint, spherical contour information is further incorporated. For the k-th frame, the observed contour center $c_{k}^{obs}=[u_{c,k}^{obs},v_{c,k}^{obs}{]}^{T}$ is obtained by extracting the sphere boundary and performing ellipse fitting. Since the sphere center is defined as the origin of the target coordinate system, its position in the camera coordinate system can be directly expressed as $C_{k}=t_{k}=[X_{k},\;Y_{k},\;Z_{k}{]}^{T}$.

Let the homogeneous image point be $x=[u,v,1{]}^{T}$, and its normalized viewing direction is

(10) $$\begin{array}{cccc} & \tilde{q}=K^{-1}x=\left[\begin{array}{c} x\\ y\\ 1 \end{array}\right] & \; & \end{array}$$

According to the tangency condition between the camera rays and the sphere surface, the contour points satisfy

(11) $$\begin{array}{cccc} & \left(C_{k}^{T}\tilde{q}{)}^{2}=\left(∥C_{k}{∥}^{2}-R^{2}\right)({\tilde{q}}^{T}\tilde{q}\right) & \; & \end{array}$$

where R denotes the sphere radius. This leads to a quadratic form describing the projected conic in the image plane

(12) $$\begin{array}{cccc} & x^{T}Q_{k}x=0 & \; & \end{array}$$

where

$$Q_{k}=K^{-T}\left(C_{k}C_{k}^{T}-\left({∥C_{k}∥}^{2}-R^{2}\right)I\right)K^{-1}$$

In the normalized image plane, the conic equation can be further expanded as

(13) $$\begin{array}{cccc} & A_{k}x^{2}+B_{k}xy+C_{k}y^{2}+D_{k}x+E_{k}y+F_{k}=0 & \; & \end{array}$$

where

$$A_{k}=R^{2}-Y_{k}^{2}-Z_{k}^{2},B_{k}=2X_{k}Y_{k},C_{k}=R^{2}-X_{k}^{2}-Z_{k}^{2},\;D_{k}=2X_{k}Z_{k},E_{k}=2Y_{k}Z_{k},F_{k}=R^{2}-X_{k}^{2}-Y_{k}^{2}$$

According to the center formula of a quadratic curve, the analytical center of the projected ellipse in the normalized image plane is given by:

(14) $$\begin{array}{cccc} & {\hat{x}}_{c,k}=\frac{X_{k}Z_{k}}{Z_{k}^{2}-R^{2}},{\hat{y}}_{c,k}=\frac{Y_{k}Z_{k}}{Z_{k}^{2}-R^{2}} & \; & \end{array}$$

which can be further mapped to the pixel plane, yielding the theoretical contour center under the current pose:

(15) $$\begin{array}{cccc} & {\hat{c}}_{k}^{ell}\left(t_{k}\right)=\left[\begin{array}{c} {\hat{u}}_{c,k}\\ {\hat{v}}_{c,k} \end{array}\right]=\left[\begin{array}{c} f_{x}{\hat{x}}_{c,k}+c_{x}\\ f_{y}{\hat{y}}_{c,k}+c_{y} \end{array}\right]=\left[\begin{array}{c} f_{x}\frac{X_{k}Z_{k}}{Z_{k}^{2}-R^{2}}+c_{x}\\ f_{y}\frac{Y_{k}Z_{k}}{Z_{k}^{2}-R^{2}}+c_{y} \end{array}\right] & \; & \end{array}$$

Based on the above analytical projection model, a contour constraint is constructed using the observed contour center $c_{k}^{obs}$ and the theoretical contour center ${\hat{c}}_{k}^{ell}(t_{k})$. This constraint is combined with the feature point reprojection error to form the joint optimization objective for the k-th frame:

(16) $$\begin{array}{cccc} & \underset{R_{k},t_{k}}{min}\sum_{i\in S_{k}}\rho \left(∥p_{i,k}-{\hat{p}}_{i,k}{∥}_{2}^{2}\right)+\beta ∥t_{k}-t_{k}^{ref}{∥}_{2}^{2}+w{∥c_{k}^{obs}-{\hat{c}}_{k}^{ell}(t_{k})∥}_{2}^{2} & \; & \end{array}$$

where ρ(⋅) denotes the Huber robust loss function, β is the weight of the translation reference term, w is the weight of the contour consistency term, and $t_{k}^{ref}$ represents the reference translation obtained from a coarse estimate. The reprojection term provides the primary pose constraint, while the contour consistency term introduces additional geometric constraints to improve stability under sparse feature conditions.

Since the above optimization problem is highly nonlinear, an iterative optimization scheme is adopted. In conventional Levenberg–Marquardt methods, the pose increment is parameterized in the Lie algebra space as an unconstrained vector $\Delta \theta \in R^{6}$, and the residual function is linearized around the current estimate $\theta^{\left(m\right)}\;$as

(17) $$\begin{array}{cccc} & r(\theta^{\left(m\right)}+\Delta \theta )\approx r(\theta^{\left(m\right)})+J^{\left(m\right)}\Delta \theta & \; & \end{array}$$

where $J^{\left(m\right)}\;$ is the corresponding Jacobian matrix. Since such methods are highly sensitive to initialization, poor initial estimates may lead to convergence to local minima, making robust initialization critical for sequential pose estimation.

Specifically, for the first frame k=0, due to the absence of temporal priors, the SQPnP algorithm is first used to obtain an initial pose $T_{0}=\{R_{0},t_{0}\}$, which is then refined using joint optimization to obtain the optimal pose $T_{0}^{*}=\{R_{0}^{*},t_{0}^{*}\}$. For subsequent frames k≥1, the optimized pose from the previous frame $T_{k-1}^{*}$ is used as the initialization for the current frame. A coarse estimate is further incorporated to construct the translation reference term $t_{k}^{ref}$, followed by iterative optimization of the joint objective.

To prevent drift caused by occlusion, rapid motion, or incorrect correspondences, a consistency check is performed after each iteration. The average reprojection error is defined as:

(18) $$\begin{array}{cccc} & e_{k}^{pt}=\frac{1}{|S_{k}|}\sum_{i\in S_{k}}{∥p_{i,k}-{\hat{p}}_{i,k}∥}_{2} & \; & \end{array}$$

When $e_{k}^{pt}$ exceeds a predefined threshold, the current estimation is considered unreliable. In this case, SQPnP is re-applied to compute a global coarse solution for the current frame, followed by iterative refinement. If the reprojection error after reinitialization is reduced, the updated result is adopted as the new candidate pose. Finally, the joint optimization is performed to obtain the optimal pose estimate for the current frame $T_{k}^{*}=\{R_{k}^{*},t_{k}^{*}\}$.

## 4. Experimental Design and Analysis

### 4.1. Simulation Experiments

To evaluate the accuracy, robustness, and stability of the proposed method, simulation experiments were conducted. A virtual pinhole camera was configured with intrinsic parameters $f_{x}=f_{y}=800$ pixels and an image resolution of 1280×720. The target-to-camera distance was set to 200 mm. The spherical target employed the 3D feature-point set constructed in the real experiments, which contains 158 feature points with a fitted radius of 73.84 mm.

Due to perspective projection effects, feature points near the spherical boundary tend to exhibit noticeable deformation in real imaging, resulting in lower localization accuracy compared to points in central regions. To improve pose estimation accuracy, a region of interest (ROI) is introduced to control the effective feature points used in the solution. Let the image resolution be W×H, and the principal point be $c=[c_{x},c_{y}{]}^{T}$. The valid imaging region is defined as a circular mask centered at the principal point, with radius:

(19) $$\begin{array}{cccc} & r_{ROI}=\lambda \cdot \frac{min(W,H)}{2} & \; & \end{array}$$

where $\left.\lambda \in (0,1\right]$ is a scaling factor that determines the size of the ROI and thus controls the number of valid feature points used for pose estimation. Table 3 presents the number of valid feature points under different ROI settings.

To further evaluate the stability of the proposed method under continuous large-range target motion, a simulation experiment with continuous pose variations was conducted. Starting from an initial pose, the target was sequentially rotated through a full revolution (360°) about the spatial X-, Y-, and Z-axes. The rotation step was set to 2°, resulting in a test sequence of 540 frames with valid observations. As reported in Table 4, the proposed method achieves high pose estimation accuracy and exhibits stable numerical behavior throughout the entire motion sequence. The mean errors of the three attitude angles remain below 0.02°, with small error dispersion and no evident cumulative drift or abrupt error jumps. These results demonstrate that the proposed method provides stable and accurate pose estimation for a spherical target undergoing continuous motion, indicating strong numerical stability and reliable tracking capability.

**Figure 8:**
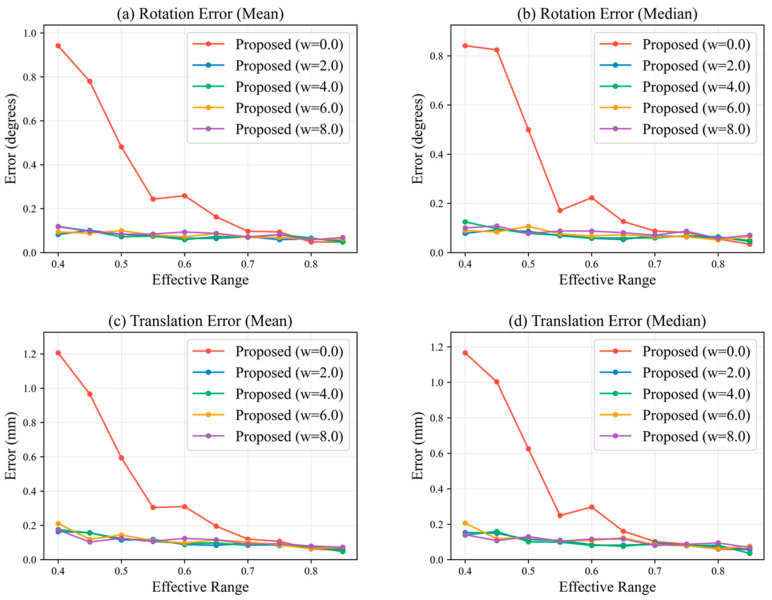
Performance comparison of the proposed method under different weight settings.

To evaluate the influence of the contour constraint weight on robustness, comparative experiments were conducted with w∈{0.0,2.0,4.0,6.0,8.0}, and rotation and translation errors were analyzed under different effective ranges. Figure 8a,b present the mean and median rotation errors, while Figure 8c,d show the mean and median translation errors (detailed data are provided in Table A1).

The results indicate that when w=0, both rotation and translation errors increase significantly under small effective ranges, suggesting that relying solely on feature point constraints makes the solution sensitive to the number and spatial distribution of points. After introducing the contour constraint, all errors decrease significantly, and the curves become smoother with respect to the effective range, indicating enhanced stability. As the weight increases, the errors gradually converge; however, when w≥6.0, further improvement becomes marginal and slight fluctuations appear in certain ranges. This suggests that excessive contour weighting may increase sensitivity to contour fitting noise and observation bias, thereby weakening the dominant role of feature point constraints. When w∈[2.0,4.0], the method achieves a good balance among accuracy, stability, and robustness. In particular, w=3.0 provides consistently lower errors across most effective ranges. For example, at an effective range of 0.85, the minimum error reaches 0.0306°, which is lower than the corresponding values under other weight settings.

To further evaluate performance under varying feature point visibility and compare with conventional PnP methods, the ROI scaling factor λ was adjusted to control the number of feature points. Gaussian noise with σ=0.3 pixels was added, and λ was varied from 0.40 to 0.85. As the effective range increased, the number of valid points gradually rose to 48. For each fixed ROI, statistics were collected over 360 consecutive frames.

**Figure 9:**
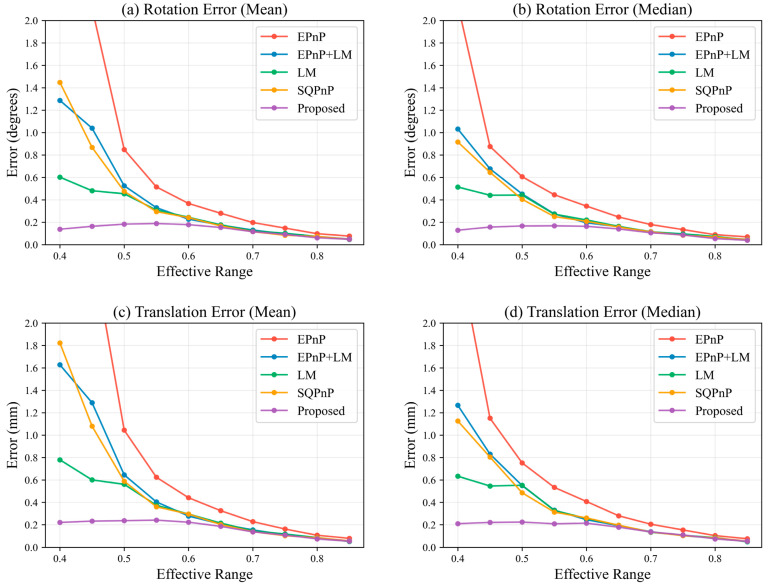
Performance comparison of different PnP algorithms under varying effective ranges.

Figure 9 presents the comparison results between the proposed method (w=3.0) and EPnP, EPnP+LM, LM, and SQPnP. Figure 9a,b show the mean and median rotation errors, while Figure 9c,d show the mean and median translation errors (detailed data are provided in Table A2). The proposed method consistently achieves superior accuracy across all ranges. In particular, under small effective ranges (λ∈[0.4, 0.55]), where the number of available points is limited and close to the minimum requirement, conventional methods become more sensitive to insufficient points, degenerate distributions, and noise, leading to larger errors. In contrast, the proposed method integrates contour center consistency constraints with feature point reprojection constraints, leveraging spherical geometric information to provide additional stability under sparse conditions and ensuring reliable pose estimation across varying numbers of feature points.

To further assess robustness against image noise, experiments were conducted with a fixed ROI ratio of λ=0.75, corresponding to 18–20 valid points. This setting effectively excludes less reliable edge points while maintaining sufficient robustness for all compared PnP methods. Zero-mean Gaussian noise with increasing standard deviation from 1.0 to 5.0 pixels was applied, forming five noise levels, and statistical analysis was performed over multiple frames at each level.

**Figure 10:**
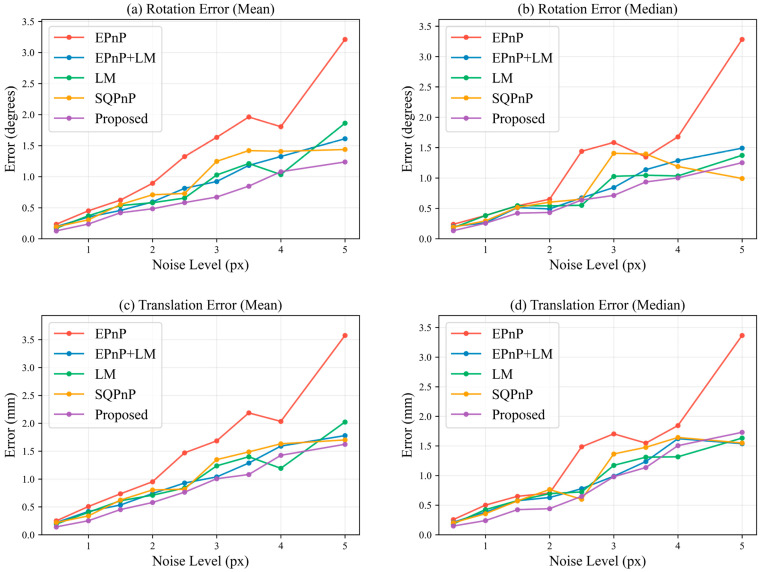
Performance comparison of different PnP algorithms under varying noise levels.

Figure 10 shows the performance comparison under different noise levels (detailed data are provided in Table A3). Figure 10a,b correspond to the mean and median rotation errors, while 10c and 10d correspond to the mean and median translation errors. Overall, as noise increases, all methods exhibit increasing errors. Although EPnP+LM, LM, and SQPnP outperform EPnP, their overall errors remain higher than those of the proposed method. In contrast, the proposed method exhibits a slower error growth rate and smoother error curves, achieving the lowest or near-lowest errors across all noise levels.

### 4.2. Real Experiments

**Figure 11:**
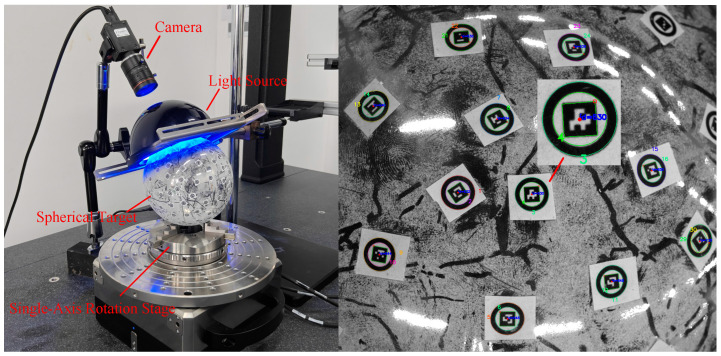
Experimental setup and marker detection results for the real-image acquisition system, where the right subfigure magnifies and displays the detection effect of the ArUco marker with ID 630, and the green numbers represent the detected ring numbers.

Based on the simulation results, the previous section has demonstrated the effectiveness and robustness of the proposed method for spherical targets. To further validate its performance under real imaging conditions, a stepwise rotation experiment was conducted using a high-precision single-axis rotation stage. In this experiment, an area-scan camera with a resolution of 5472×3648 and a pixel size of 2.4 μm×2.4 μm was used. The spherical target consisted of a real sphere with a diameter of 73.8 mm and printed feature markers attached to its surface. As shown in Figure 11, the left side presents the overall experimental setup, while the right side shows a magnified view of the actual circular marker detection results. The intrinsic parameters of the camera, obtained via Zhang’s calibration method [25], are listed in Table 5.

In the experiment, the target was rotated with an angular increment of 2°, with the *Z*-axis of the target oriented upward. The pose was gradually rotated from 0° to 90°, resulting in a total of 46 real test poses. Due to the difficulty of perfectly aligning the target coordinate system with the ideal rotation axis in practical setup, small coupled variations in roll and pitch angles are inevitably introduced during rotation.

To better analyze the rotational variation along the dominant direction, a principal-axis-based decoupling strategy is adopted. Specifically, the relative rotations between consecutive frames are first computed, and the dominant rotation axis is estimated via weighted principal direction analysis. The incremental rotations are then projected onto this fixed axis to obtain a decoupled angular sequence. The detailed procedure is provided in Algorithm A1, and the raw pose data before processing are given in Table A4.

Table 6 presents representative pose estimation results after principal-axis decoupling at 10° intervals. For each angle, the estimated roll, pitch, and yaw angles, as well as the reprojection error, are reported. As shown in the table, the reprojection error remains highly stable across the entire sequence, demonstrating the reliability and consistency of feature detection on the spherical target.

For the primary rotation angle, taking the true step angle of 2° as a reference, the relative angular increment error between consecutive frames is evaluated. The maximum error is 0.2060°, the minimum error is 0.0013°, the mean absolute deviation is 0.0552°, the root mean square error is 0.0724°, and the standard deviation is 0.0722°. These results demonstrate that the proposed method maintains high consistency and stability between successive poses and can accurately reflect the actual rotational increments of the target.

**Figure 12:**
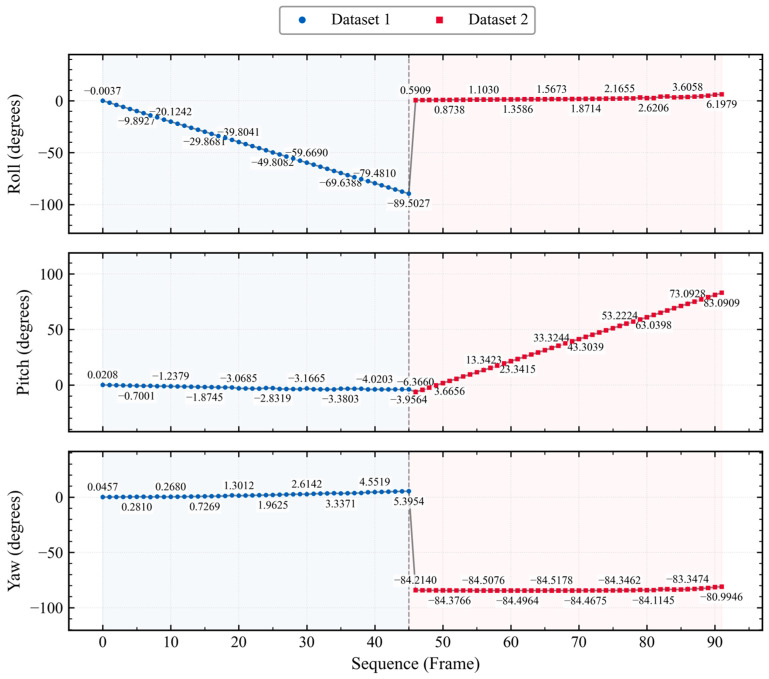
Evolution of Euler angles (roll, pitch, and yaw) over the combined sequence under two rotation configurations.

To further validate the robustness of the proposed method under continuous orientation variations, an additional experiment was conducted by sequentially altering the dominant rotation direction of the spherical target. Specifically, the target was first manually rotated with its *X*-axis serving as the primary rotation direction, and a sequence of 45 frames was collected to form the first dataset. Subsequently, the target was manually reoriented, and a second sequence was acquired with a different dominant rotation direction (approximately aligned with the *Y*-axis), resulting in a total of 91 frames. The estimated pose over the entire sequence is illustrated in Figure 12. Quantitatively, compared with the nominal step angle of 2°, the average incremental rotation error between consecutive frames is 0.0739° for the first dataset and 0.0619° for the second dataset.

As shown in Figure 12, the estimated Euler angles (roll, pitch, and yaw) exhibit smooth and continuous evolution across the entire sequence. Despite the change in the dominant rotation direction, the overall trends remain consistent with the expected motion behavior, indicating that the proposed method can maintain stable and coherent pose estimation under arbitrary orientation changes. Detailed original data from both datasets are provided in Table A6 and Table A7.

## 5. Conclusions

This paper presents a robust visual pose estimation framework for spherical cooperative targets. A unified 3D feature-point model is first constructed via multi-frame registration, followed by a joint optimization method integrating feature point reprojection error and contour center consistency constraints. The proposed approach combines SQPnP initialization, temporal iterative refinement, and Huber-based robust optimization to achieve stable and continuous pose estimation.

Experimental results demonstrate that the proposed method achieves high accuracy and robustness under various challenging conditions in simulation, including continuous pose variations, sparse feature observations, and different noise levels. In real experiments, the method shows reliable performance in both single-axis precision evaluation and multi- direction continuous rotation scenarios. The incremental consistency analysis verifies the accuracy of angular estimation under controlled rotation, while the cross- direction continuous experiment further confirms the stability and reliability of the method under varying rotation configurations.

Although the proposed method shows promising performance, its accuracy is still influenced by the geometric precision of the spherical target and the quality of feature extraction under complex imaging conditions. In future work, efforts will be directed toward improving the manufacturing and calibration accuracy of the spherical target, as well as introducing learning-based techniques to optimize feature detection and matching. These improvements are expected to further enhance the accuracy, robustness, and adaptability of the proposed method in real-world applications.

## Figures and Tables

**Table 1:** Minimum number of markers required on the spherical target under different field-of-view (FOV) sizes.

Ring Diameter *d* (mm)	FOV Side Length *L* (mm)	Effective FOV Side Length *L*′ *= L − d* (mm)	Effective FOV Area *S_FOV_* (mm^2^)	Area Coverage Ratio 4πR^2^/*S_FOV_*	Minimum Number of Markers *N* (Rounded up)
4	120	116	13,456.00	5.07	21
4	115	111	12,321.00	5.54	23
4	110	106	11,236.00	6.08	25
4	105	101	10,201.00	6.69	27
4	100	96	9216.00	7.41	30
4	95	91	8281.00	8.25	33
4	90	86	7396.00	9.23	37
4	85	81	6561.00	10.41	42

**Table 2:** Statistical analysis of overall errors in the reconstructed target point set.

Metric	Value
Total number of points (N)	158
Theoretical radius *R*_0_ (mm)	73.8
Mean radius *r* (mm)	73.8444
Mean radius error	0.0444
Fitted radius *R_f_* (mm)	73.8354
Fitted sphere center (*x_c_, y_c_, z_c_*)	(−0.0223, −0.14287, 0.1361)
Root Mean Square Error (RMSE) (mm)	0.1831
Maximum radius error (mm)	0.28917
Mean Absolute Error (MAE) (mm)	0.1617
Standard deviation (mm)	0.1782

**Table 3:** Number of effective feature points under varying ROI ranges.

ROI range	0.4	0.5	0.6	0.7	0.8	0.9	1.0
Number of points	5	7	11	16	26	48	48
Effective region radius (px)	144	180	216	252	288	324	360

**Table 4:** Statistical results of pose estimation in simulation experiments.

Metric	Mean	Median	Maximum	Minimum	Standard Deviation
Rotation error (°)	0.0309	0.0295	0.0896	0.0028	0.0149
Translation error (mm)	0.0347	0.0328	0.1129	0.0035	0.0159
Reprojection error (px)	0.3654	0.3652	0.4423	0.2942	0.0273
Roll angle error (°)	0.0191	0.0168	0.0681	0.0000	0.0146
Pitch angle error (°)	0.0174	0.0137	0.0878	0.0000	0.0140
Yaw angle error (°)	0.0077	0.0066	0.0308	0.0000	0.0059
Number of valid points	47	48	48	42	0.2478

**Table 5:** Intrinsic parameters of the camera.

Parameter	Value
Focal length (pixels)	*f_x_* = 6536.4931, *f_y_* = 6534.8233
Principal point (pixels)	(*c_x_*, *c_y_*) = (2723.6602, 1816.1003)
Radial distortion coefficients	*k_1_* = −0.0626, *k_2_* = 0.1095, *k_3_* = 0.0408
Tangential distortion coefficients	*p_1_* = 0.0011, *p_2_* = 0.0005

**Table 6:** Pose estimation results after principal-axis decoupling in the stepwise rotation experiment.

Angle (°)	Roll (°)	Pitch (°)	Yaw (°)	Reprojection Error (Pixels)
10	−0.0206	−0.0101	9.9555	0.4855
20	−0.0312	−0.0086	19.8991	0.4806
30	−0.0753	−0.0611	29.8122	0.5199
40	−0.0215	−0.1146	40.0597	0.5020
50	−0.0316	−0.1072	49.8319	0.5198
60	−0.0519	−0.1311	59.7430	0.5217
70	−0.0687	−0.1248	69.6216	0.5240
80	−0.0996	−0.1289	79.4480	0.4932
90	−0.0972	−0.1214	89.4814	0.4848

## Data Availability

The raw data supporting the conclusions of this article will be made available by the authors on request.

## Abbreviations

The following abbreviations are used in this manuscript:

PNP	Perspective-n-Point
EPNP	Efficient Perspective-n-Point
SQPNP	Sequential Quadratic Programming Perspective-n-Point
LM	Levenberg–Marquardt
DLT	Direct Linear Transformation
FOV	field-of-view
ID	Identifier
ROI	Region of Interest

## Appendix A

**Table A1:** Performance comparison of the proposed method under different weight settings.

Weighting Parameters	Value	Mean Rotation Error (°)	Median Rotation Error (°)	Mean Translation Error (mm)	Median Translation Error (mm)	Mean Reprojection Error (px)	Median Reprojection Error (px)
0	0.400	0.9418	0.8410	1.2062	1.1664	0.1943	0.1729
	0.450	0.7797	0.8245	0.9661	1.0030	0.2603	0.2473
	0.500	0.4812	0.4998	0.5940	0.6255	0.2742	0.2827
	0.550	0.2431	0.1709	0.3044	0.2489	0.3088	0.2827
	0.600	0.2589	0.2228	0.3096	0.2966	0.3098	0.2957
	0.650	0.1627	0.1261	0.1955	0.1607	0.3419	0.3556
	0.700	0.0969	0.0875	0.1198	0.1020	0.3620	0.3721
	0.750	0.0936	0.0815	0.1064	0.0877	0.3267	0.3196
	0.800	0.0480	0.0552	0.0631	0.0587	0.3435	0.3412
	0.850	0.0478	0.0342	0.0536	0.0569	0.3411	0.3312
2.0	0.400	0.0823	0.0783	0.1631	0.1526	0.2815	0.2877
	0.450	0.1009	0.0920	0.1580	0.1493	0.2843	0.2772
	0.500	0.0837	0.0859	0.1144	0.1134	0.3165	0.2943
	0.550	0.0751	0.0683	0.1183	0.1089	0.3324	0.3301
	0.600	0.0649	0.0579	0.0880	0.0840	0.3327	0.3402
	0.650	0.0645	0.0532	0.0837	0.0756	0.3221	0.3256
	0.700	0.0718	0.0695	0.0956	0.0928	0.3508	0.3584
	0.750	0.0587	0.0637	0.0821	0.0832	0.3492	0.3450
	0.800	0.0625	0.0605	0.0784	0.0721	0.3585	0.3457
	0.850	0.0533	0.0490	0.0608	0.0576	0.3703	0.3667
3.0	0.400	0.1020	0.0846	0.1572	0.1324	0.2751	0.2668
	0.450	0.1044	0.0903	0.1231	0.1400	0.3262	0.2025
	0.500	0.0834	0.0746	0.0924	0.1131	0.3074	0.2965
	0.550	0.0751	0.0640	0.1231	0.0965	0.3380	0.2994
	0.600	0.0659	0.0859	0.0924	0.0931	0.3408	0.3266
	0.650	0.0616	0.0632	0.0847	0.0744	0.3179	0.3193
	0.700	0.0634	0.0639	0.0791	0.0634	0.3379	0.3374
	0.750	0.0539	0.0734	0.0811	0.0614	0.3427	0.3451
	0.800	0.0525	0.0535	0.0600	0.0682	0.3548	0.3451
	0.850	0.0481	0.0306	0.0509	0.0448	0.3415	0.3528
4.0	0.400	0.1187	0.1247	0.1757	0.1384	0.2759	0.2608
	0.450	0.0986	0.0971	0.1552	0.1590	0.3068	0.3085
	0.500	0.0719	0.0777	0.1227	0.1010	0.3167	0.3022
	0.550	0.0749	0.0709	0.1063	0.0987	0.3020	0.3025
	0.600	0.0591	0.0589	0.0960	0.0803	0.3369	0.3245
	0.650	0.0728	0.0593	0.0968	0.0825	0.3395	0.3336
	0.700	0.0696	0.0595	0.0834	0.0848	0.3429	0.3457
	0.750	0.0816	0.0683	0.0882	0.0781	0.3593	0.3534
	0.800	0.0661	0.0640	0.0761	0.0815	0.3686	0.3742
	0.850	0.0476	0.0446	0.0474	0.0355	0.3608	0.3702
6.0	0.400	0.0936	0.0894	0.2107	0.2063	0.2782	0.2893
	0.450	0.0881	0.0838	0.1192	0.1177	0.3293	0.3111
	0.500	0.0994	0.1065	0.1436	0.1271	0.3481	0.3667
	0.550	0.0800	0.0759	0.1115	0.1054	0.3349	0.3310
	0.600	0.0718	0.0668	0.0962	0.1084	0.3193	0.3297
	0.650	0.0860	0.0725	0.1154	0.1232	0.3582	0.3543
	0.700	0.0681	0.0651	0.1031	0.0848	0.3469	0.3583
	0.750	0.0688	0.0649	0.0849	0.0793	0.3578	0.3423
	0.800	0.0600	0.0509	0.0647	0.0609	0.3547	0.3610
	0.850	0.0629	0.0647	0.0720	0.0763	0.3860	0.3725
8.0	0.400	0.1178	0.1001	0.1732	0.1409	0.2664	0.2608
	0.450	0.0962	0.1080	0.1032	0.1069	0.2825	0.2832
	0.500	0.0832	0.0776	0.1244	0.1296	0.3108	0.3043
	0.550	0.0841	0.0876	0.1071	0.1043	0.3337	0.3290
	0.600	0.0937	0.0873	0.1243	0.1157	0.3361	0.3305
	0.650	0.0871	0.0808	0.1160	0.1165	0.3409	0.3462
	0.700	0.0713	0.0707	0.0898	0.0798	0.3274	0.3397
	0.750	0.0810	0.0867	0.0927	0.0865	0.3537	0.3618
	0.800	0.0576	0.0574	0.0794	0.0942	0.3584	0.3470
	0.850	0.0685	0.0702	0.0726	0.0679	0.3586	0.3541

**Table A2:** Performance comparison of different PnP algorithms under varying effective ranges.

Algorithm	Value	Mean Rotation Error (°)	Median Rotation Error (°)	Mean Translation Error (mm)	Median Translation Error (mm)	Mean Reprojection Error (px)	Median Reprojection Error (px)
ProPosed	0.400	0.1374	0.1289	0.2206	0.2094	0.2491	0.2410
	0.450	0.1638	0.1569	0.2323	0.2207	0.2831	0.2891
	0.500	0.1830	0.1667	0.2364	0.2237	0.3045	0.2901
	0.550	0.1886	0.1681	0.2412	0.2085	0.3079	0.3067
	0.600	0.1784	0.1640	0.2227	0.2140	0.3143	0.3183
	0.650	0.1532	0.1399	0.1855	0.1780	0.3414	0.3399
	0.700	0.1158	0.1060	0.1365	0.1360	0.3373	0.3457
	0.750	0.0913	0.0869	0.1074	0.1089	0.3434	0.3409
	0.800	0.0612	0.0539	0.0744	0.0753	0.3539	0.3522
	0.850	0.0481	0.0388	0.0555	0.0544	0.3665	0.3662
EPnP	0.400	4.9876	2.1451	6.3147	2.6552	0.8565	0.4142
	0.450	2.1131	0.8761	2.6809	1.1506	0.6362	0.3175
	0.500	0.8488	0.6063	1.0443	0.7518	0.3354	0.3099
	0.550	0.5153	0.4453	0.6239	0.5335	0.3513	0.3455
	0.600	0.3676	0.3442	0.4412	0.4076	0.3530	0.3560
	0.650	0.2800	0.2467	0.3256	0.2800	0.3541	0.3520
	0.700	0.1973	0.1793	0.2277	0.2048	0.3572	0.3435
	0.750	0.1477	0.1343	0.1618	0.1535	0.3670	0.3645
	0.800	0.0979	0.0887	0.1061	0.1029	0.3693	0.3716
	0.850	0.0762	0.0695	0.0788	0.0748	0.3731	0.3686
SQPnP	0.400	1.4481	0.9165	1.8229	1.1261	0.1897	0.1964
	0.450	0.8682	0.6456	1.0801	0.8040	0.2444	0.2444
	0.500	0.4754	0.4045	0.5882	0.4852	0.2712	0.2616
	0.550	0.2947	0.2504	0.3603	0.3127	0.3075	0.3109
	0.600	0.2414	0.2082	0.2954	0.2608	0.3272	0.3214
	0.650	0.1670	0.1569	0.2020	0.1954	0.3330	0.3386
	0.700	0.1156	0.1137	0.1377	0.1350	0.3475	0.3519
	0.750	0.0838	0.0845	0.1024	0.1033	0.3528	0.3589
	0.800	0.0726	0.0661	0.0828	0.0817	0.3511	0.3503
	0.850	0.0479	0.0446	0.0568	0.0546	0.3622	0.3629
LM	0.400	0.6022	0.5143	0.7795	0.6330	0.2757	0.2640
	0.450	0.4811	0.4410	0.6002	0.5457	0.2759	0.2671
	0.500	0.4541	0.4425	0.5607	0.5522	0.2885	0.2979
	0.550	0.3087	0.2722	0.3738	0.3276	0.3128	0.3131
	0.600	0.2440	0.2202	0.2954	0.2561	0.3083	0.3103
	0.650	0.1765	0.1631	0.2145	0.1961	0.3294	0.3306
	0.700	0.1226	0.1140	0.1456	0.1326	0.3338	0.3318
	0.750	0.1016	0.0961	0.1171	0.1062	0.3491	0.3467
	0.800	0.0722	0.0726	0.0850	0.0849	0.3516	0.3531
	0.850	0.0464	0.0429	0.0519	0.0497	0.3610	0.3592
EPnP+LM	0.400	1.2873	1.0322	1.6277	1.2658	0.2074	0.2144
	0.450	1.0395	0.6769	1.2890	0.8306	0.2540	0.2484
	0.500	0.5252	0.4521	0.6452	0.5494	0.2851	0.2912
	0.550	0.3298	0.2743	0.4039	0.3302	0.3011	0.3036
	0.600	0.2273	0.1950	0.2775	0.2449	0.3202	0.3118
	0.650	0.1744	0.1601	0.2066	0.1916	0.3273	0.3373
	0.700	0.1302	0.1160	0.1539	0.1379	0.3428	0.3482
	0.750	0.0953	0.0863	0.1099	0.1027	0.3553	0.3592
	0.800	0.0728	0.0663	0.0827	0.0786	0.3545	0.3558
	0.850	0.0500	0.0467	0.0564	0.0542	0.3600	0.3600

**Table A3:** Performance comparison of different PnP algorithms under varying noise levels.

Algorithm	Noise Levels (px)	Mean Rotation Error (°)	Median Rotation Error (°)	Mean Translation Error (mm)	Median Translation Error (mm)	Mean Reprojection Error (px)	Median Reprojection Error (px)
ProPosed	0.500	0.1270	0.1332	0.1413	0.1476	0.5811	0.5716
	1.000	0.2366	0.2553	0.2515	0.2389	1.1631	1.1104
	1.500	0.4182	0.4215	0.4520	0.4242	1.7839	1.7914
	2.000	0.4824	0.4324	0.5788	0.4404	2.4161	2.3673
	2.500	0.5825	0.6382	0.7642	0.6494	2.8183	2.7959
	3.000	0.6712	0.7134	1.0052	0.9800	3.4047	3.3169
	3.500	0.8469	0.9355	1.0806	1.1352	3.9106	3.8941
	4.000	1.0797	1.0022	1.4254	1.5050	4.6072	4.6881
	5.000	1.2369	1.2523	1.6230	1.7298	5.5545	5.4308
EPnP	0.500	0.2330	0.2376	0.2491	0.2564	0.5773	0.5655
	1.000	0.4497	0.3812	0.5065	0.5003	1.2429	1.2509
	1.500	0.6222	0.5422	0.7345	0.6492	1.7282	1.6830
	2.000	0.8929	0.6503	0.9514	0.7031	2.4583	2.3976
	2.500	1.3236	1.4407	1.4700	1.4853	3.1211	3.2404
	3.000	1.6337	1.5861	1.6842	1.7042	3.5944	3.5048
	3.500	1.9606	1.3464	2.1863	1.5478	4.3982	4.2352
	4.000	1.8046	1.6777	2.0340	1.8441	5.0138	4.8646
	5.000	3.2116	3.2836	3.5763	3.3662	5.9709	5.9369
SQPnP	0.500	0.1921	0.1909	0.2189	0.2030	0.5826	0.5802
	1.000	0.3039	0.2936	0.3383	0.3555	1.2001	1.1617
	1.500	0.5507	0.5170	0.6227	0.5718	1.7349	1.7548
	2.000	0.7082	0.6017	0.8045	0.7644	2.2718	2.1717
	2.500	0.7286	0.6514	0.8167	0.5983	2.8560	2.9362
	3.000	1.2465	1.4069	1.3477	1.3648	3.3178	3.2673
	3.500	1.4196	1.3946	1.4864	1.4766	3.9522	3.9277
	4.000	1.4070	1.1895	1.6322	1.6427	4.7135	4.7566
	5.000	1.4372	0.9921	1.7011	1.5504	5.6855	5.6848
LM	0.500	0.1691	0.1809	0.1920	0.1836	0.5646	0.5595
	1.000	0.3694	0.3818	0.4017	0.4243	1.1365	1.1719
	1.500	0.5341	0.5446	0.6104	0.5757	1.7014	1.6876
	2.000	0.5801	0.5406	0.7094	0.6944	2.2265	2.1662
	2.500	0.6550	0.5512	0.8392	0.7228	2.9654	2.8346
	3.000	1.0267	1.0274	1.2362	1.1716	3.3629	3.3981
	3.500	1.2110	1.0452	1.3996	1.3097	3.8506	3.9144
	4.000	1.0339	1.0345	1.1924	1.3167	4.5121	4.5301
	5.000	1.8612	1.3741	2.0234	1.6326	5.9825	5.9974
EPnP+LM	0.500	0.1962	0.2017	0.2182	0.2113	0.6038	0.6002
	1.000	0.3487	0.2624	0.4184	0.3829	1.1281	1.1511
	1.500	0.4550	0.5129	0.5336	0.5752	1.7178	1.7188
	2.000	0.5928	0.4906	0.7395	0.6291	2.2874	2.2663
	2.500	0.8121	0.6733	0.9285	0.7781	2.9637	3.0536
	3.000	0.9216	0.8429	1.0376	0.9874	3.3264	3.2006
	3.500	1.1811	1.1373	1.2865	1.2366	3.9705	3.8385
	4.000	1.3245	1.2863	1.5908	1.6232	4.5793	4.6894
	5.000	1.6111	1.4919	1.7791	1.5383	5.6369	5.6264

## Appendix B

**Algorithm A1** Principal Axis Estimation and Rotation Decoupling
Input: rvecs_data ← a list of rotation vectors [rvx, rvy, rvz] (in radians) Output: accumulated_angles_deg ← cumulative rotation angles along the dominant axis (in degrees) 1. Initialize num_frames ← length of rvecs_data 2. If num_frames < 2, print “Error: Not enough data” and return 3. Convert each rvec in rvecs_data to rotation matrices and store in rotations 4. Initialize delta_rvecs, angles ← empty lists 5. For i ← 1 to num_frames - 1: a. Calculate relative rotation ΔR between frames i and i − 1 b. Convert ΔR to rotation vector (delta_rv) c. Compute angle = norm (delta_rv) d. If angle > 1 × 10^− 1^: i. Normalize delta_rv and append to delta_rvecs ii. Append angle to angles e. Else, append [0, 0, 0] and 0 to delta_rvecs and angles 6. Estimate dominant rotation axis u_fixed using weighted average of delta_rvecs 7. For i ← 1 to num_frames − 1: a. Compute relative rotation ΔR between frames i and i − 1 b. Project rotation onto u_fixed: delta_theta = dot (omega_i, u_fixed) c. Append delta_theta to corrected_increments 8. Compute cumulative angles: accumulated_angles = cumsum ([0] + corrected_increments) 9. Convert to degrees: accumulated_angles_deg = degrees (accumulated_angles) 10. Return accumulated_angles_deg

**Table A4:** Raw Pose Data for *Z*-axis Dominant Rotation.

Angle (°)	Roll (°)	Pitch (°)	Yaw (°)	Reprojection Error (Pixels)
0	−0.0254	0.0398	−0.0021	0.4873
2	0.0575	−0.1249	−1.8688	0.4820
4	0.1645	−0.2977	−3.9773	0.4865
6	0.2945	−0.4385	−5.9418	0.4993
8	0.3915	−0.5880	−7.8949	0.4805
10	0.5122	−0.7401	−9.9160	0.4855
12	0.6357	−0.8846	−11.8412	0.4887
14	0.7559	−1.0230	−13.8309	0.4937
16	0.8835	−1.1680	−15.8056	0.5001
18	1.0520	−1.2969	−17.8303	0.4675
20	1.1658	−1.4269	−19.8269	0.4806
22	1.3071	−1.5511	−21.8818	0.5030
24	1.5539	−1.6786	−23.8808	0.4925
26	1.7143	−1.8240	−25.8310	0.5532
28	1.8147	−1.7889	−27.7869	0.8516
30	1.9685	−1.9431	−29.7179	0.5199
32	2.1171	−2.0431	−31.7553	0.4465
34	2.3756	−2.1905	−33.8005	0.5564
36	2.5611	−2.2859	−35.8482	0.6292
38	2.7140	−2.3631	−37.7556	0.5626
40	2.9111	−2.4160	−39.9475	0.5020
42	3.0743	-2.4824	−41.7397	0.5378
44	3.2521	−2.5639	−43.7415	0.5467
46	3.3449	−2.6244	−45.7767	0.6738
48	3.6179	−2.6160	-47.6326	0.5288
50	3.7981	−2.6733	−49.7152	0.5198
52	3.9999	−2.6999	−51.6997	0.5569
54	4.1814	−2.7152	−53.6585	0.5045
56	4.3768	−2.7288	−55.6354	0.5692
58	4.5733	−2.7340	−57.6410	0.5896
60	4.7509	−2.7531	−59.6255	0.5217
62	4.9324	−2.7449	−61.5317	0.4725
64	5.1294	−2.7400	−63.6162	0.5674
66	5.3227	−2.7350	−65.5794	0.5748
68	5.5240	−2.7327	−67.5571	0.6004
70	5.6790	−2.6877	−69.5046	0.5240
72	5.8538	−2.6461	−71.4017	0.5147
74	6.0463	−2.5966	−73.4257	0.5137
76	6.2257	-2.5669	−75.3952	0.5149
78	6.4051	−2.5240	−77.3709	0.4962
80	6.5774	−2.4494	−79.3273	0.4932
82	6.7516	−2.4127	−81.3370	0.4834
84	6.9331	−2.3493	−83.3941	0.4697
86	7.1225	-2.2486	−85.4274	0.5035
88	7.2792	−2.1683	−87.4836	0.5052
90	7.4535	−2.0812	−89.3505	0.4848

**Table A5:** Decoupled Pose Data after Principal Axis Re-estimation.

Angle (°)	Roll (°)	Pitch (°)	Yaw (°)	Reprojection Error (Pixels)
0	0.0000	0.0000	0.0000	0.4873
2	0.0067	0.0063	1.8756	0.4820
4	0.0052	0.0058	3.9934	0.4865
6	−0.0117	−0.0201	5.9662	0.4993
8	−0.0172	−0.0079	7.9262	0.4805
10	−0.0206	−0.0101	9.9555	0.4855
12	−0.0197	−0.0155	11.8884	0.4887
14	−0.0267	−0.0095	13.8845	0.4937
16	−0.0212	−0.0056	15.8661	0.5001
18	−0.0255	−0.0362	17.8978	0.4675
20	−0.0312	−0.0086	19.8991	0.4806
22	−0.0378	−0.0009	21.9590	0.5030
24	−0.0114	−0.0940	23.9679	0.4925
26	0.0241	−0.0956	25.9250	0.5532
28	−0.1274	−0.0761	27.8747	0.8516
30	−0.0753	−0.0611	29.8122	0.5199
32	−0.0812	−0.0424	31.8522	0.4465
34	−0.0049	−0.1106	33.9070	0.5564
36	0.0064	-0.1171	35.9581	0.6292
38	0.0038	−0.1031	37.8669	0.5626
40	−0.0215	−0.1146	40.0597	0.5020
42	−0.0143	−0.1141	41.8537	0.5378
44	0.0038	−0.1019	43.8573	0.5467
46	−0.0293	−0.0101	45.8890	0.6738
48	−0.0387	−0.1267	47.7490	0.5288
50	−0.0316	−0.1072	49.8319	0.5198
52	−0.0327	−0.1244	51.8173	0.5569
54	−0.0457	−0.1267	53.7758	0.5045
56	−0.0492	−0.1376	55.7531	0.5692
58	−0.0558	−0.1473	57.7589	0.5896
60	−0.0519	−0.1311	59.7430	0.5217
62	−0.0618	-0.1357	61.6492	0.4725
64	−0.0629	−0.1340	63.7336	0.5674
66	−0.0538	−0.1359	65.6969	0.5748
68	−0.0326	−0.1382	67.6750	0.6004
70	−0.0687	−0.1248	69.6216	0.5240
72	−0.0824	−0.1289	71.5191	0.5147
74	−0.0912	−0.1390	73.5440	0.5137
76	−0.0824	−0.1273	75.5137	0.5149
78	−0.0791	−0.1206	77.4901	0.4962
80	−0.0996	−0.1289	79.4480	0.4932
82	−0.0833	−0.1047	81.4581	0.4834
84	−0.0787	−0.0977	83.5166	0.4697
86	-0.0911	−0.1240	85.5534	0.5035
88	−0.1045	−0.1046	87.6109	0.5052
90	−0.0972	−0.1214	89.4814	0.4848

**Table A6:** Raw Pose Data for *X*-axis Dominant Rotation.

Angle (°)	Roll (°)	Pitch (°)	Yaw (°)	Reprojection Error (Pixels)
0	−0.0037	0.0208	0.0457	2.6768
2	−1.8626	−0.1226	0.0940	2.6467
4	−4.0310	−0.2856	0.1270	2.6496
6	−5.9142	−0.4189	0.1669	2.6677
8	−7.9569	−0.5642	0.2232	2.6625
10	−9.8927	−0.7001	0.2810	2.6689
12	−11.9553	−0.8412	0.3606	2.6621
14	−14.0710	−0.7913	0.0307	2.5454
16	−15.9361	−1.1055	0.4926	2.6712
18	−18.1942	−1.0903	0.1800	2.5379
20	−20.1242	−1.2379	0.2680	2.5638
22	−22.1439	−1.3710	0.3420	2.5692
24	−24.0254	−1.4832	0.4066	2.5674
26	−26.0488	−1.6287	0.5265	2.5644
28	−27.9396	−1.7543	0.6091	2.6006
30	−29.8681	−1.8745	0.7269	2.6020
32	−31.9084	−1.9767	0.8168	2.5606
34	−33.9399	−2.0940	0.9297	2.5848
36	−35.8003	−2.1985	1.0601	2.5817
38	−37.7015	−2.2819	1.6110	2.6721
40	−39.8041	−3.0685	1.3012	2.3700
42	−41.7600	−3.1752	1.4456	2.3904
44	−43.6912	−3.2705	1.5868	2.3763
46	−45.6738	−3.3501	1.7406	2.3884
48	−47.6939	−2.7474	1.8352	2.6233
50	−49.8082	−2.8319	1.9625	2.5705
52	−51.7126	−3.5821	2.1951	2.3935
54	−53.7609	−3.6540	2.3680	2.4003
56	−55.6315	−3.7216	2.5342	2.4041
58	−57.7409	−3.7992	2.6972	2.4174
60	−59.6690	−3.1665	2.6142	2.6284
62	−61.5650	−3.8720	3.0155	2.4112
64	−63.5845	−3.9294	3.2096	2.4303
66	−65.5856	−3.9437	3.3711	2.4057
68	-67.6503	−3.9875	3.5417	2.4248
70	−69.6388	−3.3803	3.3371	2.6443
72	−71.6940	−3.4025	3.5059	2.6399
74	−73.6299	−3.4287	3.6443	2.6440
76	−75.5535	−3.4474	3.7820	2.6521
78	−77.4772	−4.0293	4.4021	2.4303
80	-79.4810	−4.0203	4.5519	2.4318
82	−81.4769	−4.0284	4.7292	2.4459
84	−83.5194	−4.0244	4.9031	2.4564
86	−85.4997	−3.9842	5.0658	2.4495
88	−87.5682	−3.9548	5.2503	2.4551
90	−89.5027	−3.9564	5.3954	2.4361

**Table A7:** Raw Pose Data for *Y*-axis Dominant Rotation.

Angle (°)	Roll (°)	Pitch (°)	Yaw (°)	Reprojection Error (Pixels)
0	0.5909	−6.3660	−84.2140	1.8945
2	0.6774	−4.4609	−84.2572	1.9117
4	0.7119	−2.3936	−84.2885	1.8973
6	0.7789	−0.3746	−84.3271	1.8855
8	0.8625	1.6611	−84.3595	1.7790
10	0.8738	3.6656	−84.3766	1.8852
12	0.9550	5.5110	−84.4152	1.7696
14	0.9566	7.6456	−84.4409	1.8870
16	1.0054	9.4984	−84.4619	1.8717
18	1.1044	11.4304	−84.4948	1.7663
20	1.1030	13.3423	−84.5076	1.8576
22	1.1633	15.3459	−84.5167	1.8595
24	1.2219	17.4319	−84.5384	1.7683
26	1.2813	19.3588	−84.5570	1.7519
28	1.3325	21.4070	−84.5664	1.7474
30	1.3586	23.3415	−84.4964	1.7826
32	1.4476	25.3766	−84.4824	1.7343
34	1.4647	27.4390	−84.4914	1.8261
36	1.4911	29.2898	−84.5084	1.8497
38	1.5350	31.2949	−84.5044	1.8410
40	1.5673	33.3244	−84.5178	1.8556
42	1.5490	35.3273	−84.5046	1.9222
44	1.5672	37.4554	−84.5266	1.9639
46	1.6857	39.3470	−84.4935	1.8920
48	1.7695	41.2949	−84.4734	1.8890
50	1.8714	43.3039	−84.4675	1.7951
52	1.8539	45.2505	−84.4620	1.8813
54	1.9463	47.2628	−84.4261	1.8734
56	2.0546	49.1850	−84.3711	1.8321
58	2.0667	51.1426	−84.4026	1.8808
60	2.1655	53.2224	−84.3462	1.8547
62	2.3604	55.2343	−84.2654	1.7369
64	2.3395	57.0967	−84.2685	1.8580
66	3.1315	59.0032	−83.8848	1.8655
68	2.5981	61.0076	−84.1690	1.7725
70	2.6206	63.0398	−84.1145	1.8547
72	3.9582	65.1093	−83.3869	2.3359
74	4.1541	67.0797	−83.2742	2.4658
76	3.2605	69.1941	−83.6785	1.7622
78	3.4544	71.1689	−83.5332	1.7552
80	3.6058	73.0928	−83.3474	1.8396
82	3.9653	75.0900	−83.0136	1.8394
84	4.3771	77.1300	−82.6427	1.8460
86	4.9239	79.0339	−82.1620	1.8492
88	5.8530	81.1201	−81.3504	1.7554
90	6.1979	83.0909	−80.9946	1.6951
